# Potency classification of isothiazolinone compounds based on defined approaches of skin sensitization in OECD GL 497

**DOI:** 10.5620/eaht.2023026

**Published:** 2023-12-19

**Authors:** Hyejin Kim, Juyoung Park, Handule Lee, Jinseon Son, Yeonjung Park, Heekyung Bae, Sun-Young Park, Sang Hee Lee, Jungkwan Seo, Sunkyung Shin, Kwangsik Park

**Affiliations:** 1 College of Pharmacy, Dongduk Women’s University, Seoul, Republic of Korea; 2 TO21 Co., Ltd, 350, Seocho-daero, Seocho-gu, Seoul, Republic of Korea; 3 Environmental Health Research Department, National Institute of Environmental Research, Ministry of Environment, Incheon, Republic of Korea

**Keywords:** Skin Sensitization, Defined Approaches, Isothiazolinone

## Abstract

Regulatory decisions for skin sensitization are now based on adverse outcome pathway (AOP) and integrated approaches to testing and assessment (IATA). Based on these, Organisation for Economic Co-operation and Development (OECD) guidelines on defined approaches for skin sensitization were adopted with a fixed data interpretation procedure (DIP). In the guidelines, “Defined Approaches” (DA) on skin sensitization uses the results from multiple information sources of *in chemico, in vitro*, and *in silico* data to achieve an equivalent predictive capacity as those of the animal tests. In this review, we evaluated the skin sensitization of eleven isothiazolinone compounds including 4,5-Dichloro-2-octyl-3(2H)-isothiazolone (DCOIT), 2-n-Octyl-4-isothiazolin-3-one (OIT), 2-Methyl-4-isothiazolin-3-one (MIT), 1,2-Benzisothiazolin-3-one (BIT), 1,2-Benzisothiazolin-3-one, 2-butyl (BBIT), 5-Chloro-2-methyl-4-isothiazolin-3-one (CMIT), 2-methyl-4,5-trimethylene-4-isothiazolin-3-one (MTMIT), 2-methyl-1,2-benzothiazol-3-one (MBIT), 2-methyl-1,2-benzothiazole-3-thione (MBIT-S), 1,2-benzisothiazolin-3-one, 2-methyl-, 1,1-dioxide (BBIT-O), and a mixture of CMIT/MIT. Data from direct peptide reactivity assay (DPRA), human cell line activation (h-CLAT) test, and quantitative structure activity relationship (QSAR) Toolbox were evaluated and were applied to the DIP to derive a prediction of hazard identification and a potency classification. Among the evaluated chemicals, six isothiazolinone compounds were classified to be UN GHS 1A, one compound to be UN GHS 1, and four compounds could not be classified due to lack of data. The results of sensitizer chemicals were found to coincide well with those of *in vivo* test.

## Introduction

The adverse outcome pathway (AOP) is a conceptual framework that systematically organizes available data and knowledge that describes scientifically plausible relationships across multiple levels of biological organization between a molecular initiating event (MIE) and subsequent key events (KEs), culminating in an adverse outcome (AO) [1]. Although there are still challenges and limitations in applying AOPs to ascertain chemical safety, the uses of the AOP framework have extended to data integration and guiding chemical use decision-making. With the global interest in promoting the welfare of the animals in chemical testing, the regulatory requirements to find alternative test methods have expanded and the needs for evaluation of all existing data to determine chemical safety without animal testing have increased [2]. Based on the trends, the concept of integrated approaches to testing and assessment (IATA) which integrates the results of in vitro/in silico/in chemico data, has been developed. Some elements within IATA are flexible approaches so that the organization and assessment of data such as weight of evidence (WoE) are determined by expert judgment. However, some elements, like test guidelines, within an IATA are standardized. In the standardized part of IATA, data can be evaluated using fixed data interpretation procedures (DIP) [3-5]. With the standardizing information sources and DIP, the expert judgment for the decision-making of chemical safety would not be necessary because a decision tree can be used in the evaluation, a concept called a “Defined Approaches” (DA). Because DA uses more than one assay system and combines the results, it can reduce the limitations of in vitro tests to extrapolate animal studies to human health studies. AOP can support the development of DA, which provides acceptable regulatory tools without animal testing [6,7]. Recently, Organisation for Economic Co-operation and Development (OECD) developed and published the guidelines for DA methods on skin sensitization, which use the results from multiple information sources to achieve an equivalent or better predictive capacity than that of the animal tests. Data from direct peptide reactivity assay (DPRA), human cell line activation test (h-CLAT), KeratinoSens™ test, and data obtained by using quantitative structure activity relationship (QSAR) Toolbox were applied to the DIP to derive a prediction without the need for expert judgment [8, 9].

Isothiazolinone compounds are organic compounds with five-membered heterocyclic rings and are one of the most widely used biocides or preservatives to prevent bacterial and fungal growth. They are used in various household and industrial products, such as emulsion paints, wood varnishes, adhesives, and natural and artificial leather. It is known that their biocidal mechanism is through the interaction of nitrogen–sulfur (N–S) bonds of the isothiazolinone ring with the thiol groups (R–SH) of the membrane proteins of the micro-organisms. The oxidized thiol group leads to free radical generation and the dysfunction of the pivotal proteins, which can result in microbial cell death. Furthermore, isothiazolinone biocides inhibit microbial growth and metabolism by interfering with the mitochondrial production of adenosine triphosphate (ATP). However, a few isothiazolinone compounds including 5-Chloro-2-methyl-4-isothiazolin-3-one (CMIT), 2-Methyl-4-isothiazolin-3-one (MIT), and other structural derivatives are reported to have a weak, moderate, or strong sensitizing potential based on animal assays like the guinea pig maximization test (GPMT) or local lymph node assay (LLNA) [10-12].

In this study, we evaluated data of eleven isothiazolinone compounds for skin sensitization using DA of ITSv2 in OECD GL 497. The eleven isothiazolinone compounds were 4,5-Dichloro-2-octyl-3(2H)-isothiazolone (DCOIT), 2-n-Octyl4-isothiazolin-3-one (OIT), 2-Methyl-4-isothiazolin-3-one (MIT), 1,2-Benziso thiazolin-3-one (BIT), 1,2-Benzisothiazolin-3-one, 2-butyl (BBIT), 5-Chloro-2-methyl-4-isothiazolin-3-one (CMIT), 2-methyl-4,5-trimethylene-4-isothiazolin-3-one (MTMIT), 2-methyl-1,2-benzothiazol-3-one (MBIT), 2-methyl-1,2-benzothiazole-3-thione (MBIT-S), 1,2-benzisothiazolin-3-one, 2-methyl-, 1,1-dioxide (BBIT-O), and a mixture of CMIT/MIT.

## Defined approaches (ITSv2) for skin sensitization

### Chemicals for DASS

The evaluated chemicals with CAS numbers, molecular weight, simplified molecular input line entry system (SMILES), structural information, and physicochemical information such as Lop P are described in Table 1. SMILES is a chemical notation that allows a user to represent a chemical structure in a way that can be used by the computer, and it is necessary for the OECD QSAR Toolbox.

### *In chemico* and *in vitro* data

A systematic electronic search of the literature on the physicochemical information,* in chemico*, *in silico*, *in vitro*, and *in vivo* data of the isothiazolinone compounds was done in PubMed, and other science direct databases, and the reports from OECD, National Institute of Environmental Health Science (NIEHS), and other institutes. DA in OECD GL 497 needs* in chemico*, *in vitro*, and *in silico* data. Similar to* in chemico* data, direct peptide reactivity assay (DPRA) assesses the ability of a substance to form a hapten-protein complex, which is KE1 in the skin sensitization AOP [13]. There are two *in vitro* assay tests; KeratinoSensTM and human cell line activation test (h-CLAT). KeratinoSensTM assesses the ability of substances to activate and induce the expression of cyto-protective genes in keratinocytes based on activation of the Keap1-Nrf2 pathway of KE2 in AOP [14]. h-CLAT assesses the ability of substances to activate and mobilize dendritic cells in the skin based on KE3 in the AOP [15].

### *In silico* data: OECD QSAR Toolbox for ITSv2

Existing data of QSAR Toolbox (QSAR TB) were obtained by a previously described process of data collection. When the existing compound evaluation data were not available, OECD QSAR Toolbox v.4.5 (QSAR TB) with the automated workflow was run by the authors as described in the protocol in GL 497. QSAR TB is a freely available software application that supports reproducible and transparent chemical hazard assessment by offering functionalities for retrieving experimental data, stimulation metabolism, and profiling properties of chemicals [9]. The results obtained from the TB were used as the *in silico* information source for the ITSv2.

### Data interpretation procedure

For the hazard assessment and potency categorization of isothiazolinone compounds, the ITSv2 uses the scores assigned to the quantitative results from the DPRA, h-CLAT, and QSAR TB, which allocates the chemicals into UN GHS category 1A (strong sensitizer), category 1B (other sensitizer) or Not Classified (nonsensitizer). A simple score-based system and potency classification of OECD GL 497 are shown in Table 2.

**Figure 1.**
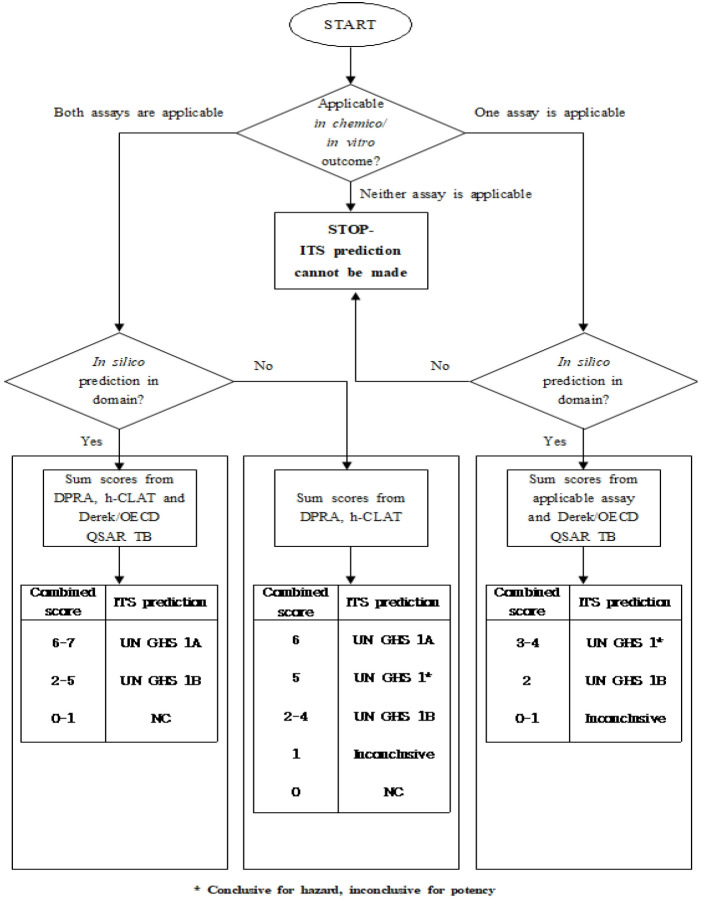
Decision tree for assigning confidence to the ITS DA predictions.

Depending on the applicability of the individual information sources, three different scenarios for the ITSv2 are possible as shown in Fig.1. In Scenario 1, all three information sources are applicable. In Scenarios 2 and 3, only two information sources are applicable. Where all three information sources are applicable, a conclusive ITS prediction can be made. In some cases, when there are only two information sources are applicable, a conclusive ITS prediction can also be made [9].

### Hazard assessment and potency classification of isothiazolinone compounds

DASS of ITSv2 in OECD GL 497 requires information from DPRA, h-CLAT, and QSAR TB, and can identify hazards to discriminate between sensitizers and nonsensitizers. Furthermore, it can also classify skin sensitizers into subcategories such as GHS 1A and 1B classification. Data for DPRA, h-CLAT, and QSAR were obtained from the published references and were reviewed in this study (16-22). Some of the QSAR data which have not been published before were generated in this study by using QSAR TB. The* in chemico*, *in vitro*, and *in silico* data and references are shown in Table 3. Some of the chemicals have been found to have reliable data of DPRA, h-CLAT, and QSAR TB as shown in Table 3. The data could be scored according to the simple score-base system depending on assays from OECD TG 442E and 442C, and an *in silico* QSAR TB, which is shown in Table 2. If the mean value of cysteine and lysine % depletion is 42.47% and over, the score is 3. When MIT value of h-CLAT is 10 and less, the score is 3. When positive results are obtained by QSAR TB, the score is 1. Based on the scoring system and decision tree (Fig.1), the total scores and potential categorization were assessed and the results are shown in Table 4.

As shown in Table 4, the total score of DCOIT, OIT, MIT, BIT, BBIT, and CMIT/MIT ranged from 6 ~ 7 and they were classified as GHS 1A, which means strong sensitizers. However, total scores of CMIT were 4 due to a lack of h-CLAT data and classified as GHS 1. In the decision tree presented in Fig.1, GHS 1 means that the hazard identification is conclusive, but the potency classification is not conclusive. In the case of MTMIT, MBIT, MBIT-S, and BBIT-O, no information on the DPRA, h-CLAT, and QSAR TB was found. Although, in this study, the QSAR TB for the prediction was run and results were obtained, the hazard identification and/or potency classification could not be evaluated with only the QSAR data.

For the validation or verification of the results of DASS, the results were compared to the *in vivo* data of local lymph node assay (LLNA) and Guinea pig maximization test (GPMT) (16, 18, 23-27). In LLNA, the results were regarded as positive when the stimulation index (SI) was ≥3. SI is a value calculated to assess the skin sensitization potential of a test substance, which is the ratio of the proliferation in treated groups to that in the concurrent vehicle control group. In GPMT, the Magnusson and Kligman grading scale is applied for the grading of skin sensitization results, depending on the formation of erythema. When the *in vivo* data were compared to those of DASS, the results of DASS were very similar to the results of the *in vivo* as shown in Table 4.

## Figures and Tables

**Table 1.** Information of isothiazolinone compounds for the evaluation by defined approaches (DA) on skin sensitization.

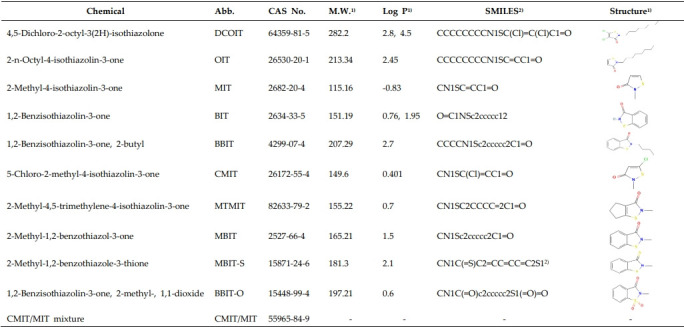

1) Information of molecular weight, Log P, and structure was obtained from PubChem.

2) SMILES data were available from OECD QSAR TB (MBIT-S was obtained from PubChem). SMILES; Simplified Molecular Input Line Entry System

**Table 2.** Schematic of the ITS defined approaches (DA).

Score	h-CLAT MIT μg/mL	DPRA Mean Cysteine and Lysine% depletion	DPRA Cysteine% depletion	*In silico* (ITSc1: DEREK; ITSv2: OECD TB)
3	≤10	≥42.47	≥98.24	
2	>10, ≤150	≥22.62, <42.47	≥23.09, <98.24	
1	>150, ≤5000	≥6.38, <22.62	≥13.89, <23.09	Positive
0	Not calculated	<6.38	<13.89	Negative
				
	Potency	Total Battery Score		
	UN GHS 1A	6-7		
	UN GHS 1B	2-5		
	Not classified	0-1		

The DA is a simple score-based system depending on assays from OECD TG 442E and 442C, and an in silico structure-based prediction. ITS; Integrated testing strategies, h-CLAT; Human cell line activation test, DPRA; Direct peptide reactivity assay.

**Table 3.** Results of DPRA, h-CLAT, and QSAR TB for the isothiazolinone compounds.

Chemical	DPRA			h-CLAT			QSAR TB		Ref.
	Cys. (%)	Lys. (%)	Ave. (%)	CD86 (EC150)	CD54 (EC200)	MIT	Domain	Result	
4,5-Dichloro-2-octyl-3(2H)-isothiazolone (DCOIT)	100	10.4	55.2	NA	0.92	0.92	-	P	[16, 17, 18]
2-n-Octyl-4-isothiazolin-3-one (OIT)	100	0	50	7.26	0.95	0.95	-	P	[16, 17, 18]
2-Methyl-4-isothiazolin-3-one (MIT)	100	0	50	11.8	11.6	11.6	-	P	[16, 17, 18]
1,2-Benzisothiazolin-3-one (BIT)	97.7	9.7	53.7	-	0.54	0.54	-	P	[19, 20, 21]
1,2-Benzisothiazolin-3-one, 2-butyl (BBIT)	100	0	50	3.15	3.01	3.01	-	P	[16, 17, 18]
5-Chloro-2-methyl-4-isothiazolin-3-one (CMIT)	96.3	35.1	65.7	-	-	-	-	P	[18, 19]
2-Methyl-4,5-trimethylene-4-isothiazolin-3-one (MTMIT)	-	-	-	-	-	-	In^1)^	P^1)^	-
2-Methyl-1,2-benzothiazol-3-one (MBIT)	-	-	-	-	-	-	In^1)^	P^1)^	-
2-Methyl-1,2-benzothiazole-3-thione (MBIT-S)	-	-	-	-	-	-	NA^1)^	NA^1)^	-
1,2-Benzisothiazolin-3-one, 2-methyl-, 1,1-dioxide (BBIT-O)	-	-	-	-	-	-	In^1)^	P^1)^	-
CMIT/MIT mixture (CMIT/MIT)	90.9	4.3	47.6	2.21	-	2.21	-	P	[18, 20, 22]

1) QSAR TB results were obtained by directly running the OECD Toolbox program in this laboratory.

MIT; Minimum Induction Threshold [min (EC150 CD86, EC200 CD54), P; Positive, NA; QSAR results were not obtained due to data limitation, In; In domain.

**Table 4.** Scores obtained by the results of DPRA, h-CLAT, and QSAR TB.

Chemical	Score			Total Score	Classification	*In vivo*	
	DPRA	h-CLAT	QSAR TB			Result	Ref.
4,5-Dichloro-2-octyl-3(2H)-isothiazolone (DCOIT)	3	3	-	6	UN GHS 1A	Sensitizer	[23]
2-n-Octyl-4-isothiazolin-3-one (OIT)	3	3	1	7	UN GHS 1A	Sensitizer	[24]
2-Methyl-4-isothiazolin-3-one (MIT)	3	2	1	6	UN GHS 1A	1A	[16, 18, 25]
1,2-Benzisothiazolin-3-one (BIT)	3	3	1	7	UN GHS 1A	Sensitizer	[16, 18, 25, 26]
1,2-Benzisothiazolin-3-one, 2-butyl (BBIT)	3	3	1	7	UN GHS 1A	-	-
5-Chloro-2-methyl-4-isothiazolin-3-one (CMIT)	3	-	1	4	UN GHS 1	Sensitizer	[26]
2-Methyl-4,5-trimethylene-4-isothiazolin-3-one (MTMIT)	-	-	1	1	-	Sensitizer	[26]
2-Methyl-1,2-benzothiazol-3-one (MBIT)	-	-	1	1	-	Sensitizer	[27]
2-Methyl-1,2-benzothiazole-3-thione (MBIT-S)	-	-	-	-	-	-	-
1,2-Benzisothiazolin-3-one, 2-methyl-, 1,1-dioxide (BBIT-O)	-	-	1	1	-	-	-
CMIT/MIT mixture (CMIT/MIT)	3	3	1	7	UN GHS 1A	1A	[16, 18, 25]

-; This means that there were no results available or that classification is not possible
